#  Open science in Spain: Influence of personal and contextual factors on deposit patterns

**DOI:** 10.12688/f1000research.160207.1

**Published:** 2025-02-14

**Authors:** Daniel de Gracia Palomera

**Affiliations:** 1 Centre d’Estudis Sociològics sobre la Vida Quotidiana i el Treball (QUIT), Institut d’Estudis del Treball., Universitat Autònoma de Barcelona Departament de Sociologia, Bellaterra, Catalonia, Spain

**Keywords:** Open Science, Data Sharing, Academic Repositories, Deposit Practices, Institutional Policies.

## Abstract

**Background:**

This study investigates factors influencing the deposit of academic publications and research data in open access repositories by Spanish researchers.

**Methods:**

Using survey data from a sample of Spanish academics, the research examines the impact of personal attributes (e.g., gender, age, knowledge of open science) and contextual variables (e.g., academic discipline, institutional type) on deposit behaviours. Quantitative methods, including chi-square tests and regression analysis, reveal significant associations between knowledge of open science and deposit practices.

**Results:**

Researchers familiar with open science principles were more likely to deposit multiple versions of articles and datasets, albeit with varying intensity. Key findings highlight disciplinary and institutional differences: researchers in Life Sciences and Experimental Sciences showed higher engagement with both article and data deposits, whereas Health Sciences lagged. Gender differences were also observed, with male researchers depositing articles and datasets more frequently than their female counterparts, though age showed limited impact. Public institutions exhibited lower data deposit rates despite mandates supporting open access.

**Conclusions:**

The study underscores the need for tailored policies, including awareness campaigns, infrastructure investment, and discipline-specific strategies, to promote equitable and widespread adoption of open science practices. Findings contribute to understanding open science implementation, emphasizing the interplay of individual, institutional, and systemic factors.

## Introduction

With the growing importance of open science, sharing of and access to scholarly production through open repositories and scholarly social networks has become increasingly relevant ( MacIntyre et al., 2015; Quinn, 2023). These channels allow researchers to share their work in different versions: pre-prints (unreviewed versions), accepted revised versions, and final published versions. However, their actions, as well as the choice of deposit route and version type can vary considerably, influenced not only by licensing restrictions, but also by personal (gender and age) and contextual characteristics such as discipline, prior knowledge about open science, and the type of institution in which you work ( Miguel et al., 2018; Perrier et al., 2020).

Understanding deposit patterns based on these factors is crucial for institutions and agencies that promote open access, as it allows identifying which factors are associated with more and less frequent deposit practices ( Ishak et al., 2019). In addition, it allows us to assess whether there are structural or individual barriers that limit the use of open repositories and academic social networks ( Perrier et al., 2020).

In the Spanish context, in which this research is framed, access to and deposit of the results of publicly funded research is regulated by the Law on Science, Technology and Innovation 17/2022, of 5 September, amending Law 14/2011 and by the mandates of the Horizon 2020 Programme (art. 29.2). This legislative framework establishes the obligation to guarantee public access both to scientific publications and, according to the Horizon Programme, to the data generated during research, promoting transparency, reuse and dissemination of knowledge.

Researchers wholly or partially funded by public funds are obliged to comply with these mandates. The requirements can also be complemented by specific policies from universities, regional governments or funders, which often include detailed guidelines to ensure access to research data. This ensures that scientific results and underlying data are widely disseminated for the benefit of the scientific community and society ( Políticas y Mandatos: España|Recolecta, n.d.).

As for scientific publications, researchers in Spain must deposit the final version in institutional or thematic repositories accessible to the public. This obligation is supported by Article 37 of the Law on Science, Technology and Innovation, which establishes a maximum period of 12 months from publication, and by Article 29.2 of the Horizon 2020 Programme, which qualifies this period to 6-12 months depending on the specifications of the funded project. The deposit can be made through open access journals (gold or hybrid models) or in commercial journals, if the publisher’s embargo policy is respected.

Regarding research data, these must be managed and deposited following the FAIR (Findable, Accessible, Interoperable, Reusable) principles, which is an explicit requirement of the Horizon 2020 Open Science Pilot Plan. The aim is to ensure that the datasets generated during funded projects are accessible to the scientific community and the public, unless there are justified reasons such as confidentiality, security or intellectual property rights. This measure is also supported by Article 37 of Science Law, which emphasizes the obligation to guarantee public access to the data that underpins scientific publications.

The repositories used for the deposit must be recognized by the scientific community, whether institutional, thematic or specific open access platforms. Data may include raw or processed sets and should be accompanied by detailed metadata for easy location and reuse. In the case of projects funded by the Horizon 2020 Programme, researchers are required to develop a Data Management Plan, which details how data will be collected, preserved and shared throughout the project, meeting the deadlines set out in the project.

Compliance with these regulations ensures scientific transparency, collaboration in the reuse of data, and guarantees that citizens have access to the results and data of research funded with public resources, generating a positive impact on the advancement of knowledge and the social return on public investments. Spanish and European legislation converges in this effort to consolidate an open science system at the service of society.

In short, in the current Spanish context, open access regulations have configured a mandatory framework for the deposit of publications and research data, establishing deadlines, permitted versions and appropriate types of repositories. However, compliance with these requirements depends not only on the existence of such regulations, but also on personal and contextual factors that influence researchers’ decisions when selecting depository versions and platforms ( Miguel et al., 2018; Perrier et al., 2020). These decisions can vary significantly depending on knowledge of the mandates, academic discipline, or institutional policies that govern their environment.

The actual patterns of deposit in institutional or thematic repositories show differences that may be related to aspects such as the age of the researchers, their previous experience with open access, or the specific constraints and opportunities of the disciplines in which they work ( Miguel et al., 2018; Nicholas et al., 2012). Added to this are organizational and technological factors, such as the support offered by academic institutions or the limitations imposed by publishers, which influence the effective implementation of open access ( Bratt, 2023a, 2023b; Mbughuni et al., 2022).

This study aims to analyse some of these patterns of deposit of articles and research data, based on personal variables (gender, age, knowledge about open science) and contextual variables (discipline, type of centre). It also seeks to identify associations between these characteristics and the preference for a specific version type and deposit platform.

## Literature review

The advancement of open science practices has fundamentally transformed scholarly communication patterns over the past decade. The proliferation of open access repositories and academic social networks has created multiple pathways for researchers to share their work, leading to complex deposit behaviours influenced by various personal and institutional factors ( Boudry & Bouchard, 2017; Xia, 2007). This literature review examines the current understanding of researchers’ deposit practices and the factors influencing their choices.

The development of institutional and subject repositories has provided researchers with formal channels for sharing their work openly. Studies have shown that repository usage has grown significantly, though adoption rates vary considerably across disciplines and institutions ( Quinn, 2023). The introduction of institutional repositories marked a significant shift in scholarly communication, offering researchers structured platforms for long-term preservation and dissemination of their work. These systems have evolved from basic document storage solutions to sophisticated platforms supporting various types of research outputs and versions ( Bratt, 2023b; Ishak et al., 2019).

The coexistence of different versions of scholarly works - preprints, accepted manuscripts, and published versions - has created a complex ecosystem of sharing practices. Previous studies have documented how researchers navigate these options based on publisher policies, institutional mandates, and personal preferences ( Miguel et al., 2018). Research has revealed that the timing of deposits significantly influences citation impact and research visibility, with early sharing through preprints potentially accelerating scientific discourse and increasing citation rates ( Borrego, 2017). Furthermore, studies have shown that researchers’ version-sharing decisions are often influenced by a complex interplay of factors, including copyright restrictions, institutional policies, and disciplinary norms ( Asadi et al., 2019; Haddow, 2008; Miguel et al., 2018; Quinn, 2023).

A comprehensive understanding of these variables is essential for designing strategies to enhance the adoption of deposit practices and facilitate equitable access to academic outputs.

### Disciplinary and institutional influences

The emergence of repositories and academic social networks has introduced new dynamics in scholarly communication, fundamentally altering how researchers disseminate their work. Academic social networks have gained substantial popularity due to their intuitive interfaces and integrated networking features, though their role in the broader open science ecosystem remains a subject of debate ( Borrego, 2017; Boudry & Bouchard, 2017). Research has indicated that platform choice often reflects deeper patterns in scholarly communication practices, with different platforms serving distinct purposes in researchers’ dissemination strategies ( Borrego, 2017).

The influence of academic discipline on deposit practices in repositories is one of the most significant studied variables affecting researcher behaviour. Scholars from the Exact Sciences and Natural Sciences, for example, are far more likely to utilize academic social networks such as ResearchGate compared to those from the Humanities and Social Sciences. This discrepancy may be attributed to the differing norms and traditions across fields, with the former disciplines placing a higher emphasis on rapid dissemination of research findings and collaborative visibility. In contrast, researchers in the Humanities and Social Sciences often rely on longer publication cycles and may view repositories as secondary to traditional publishing methods ( Larivière et al., 2006; Stark et al., 2020). In contrast, other past articles ( Haddow, 2008) denied this disciplinary variance, arguing that institutional policies is the latent variable that causes deposit differences and not discipline cultures.

Institutional affiliation is another critical determinant. Researchers aligned with institutions that actively promote open access and provide structured support for depositing research outputs are more likely to engage with these platforms ( Asadi et al., 2019; Bratt, 2023b). Notably, some institutions exhibit higher participation rates in academic social networks than in their institutional repositories. This trend may reflect a perception that academic social networks offer greater visibility and networking opportunities, or it may indicate a lack of sufficient infrastructure or advocacy for institutional repository use ( Asadi et al., 2019). These observations underscore the necessity of fostering institutional cultures that prioritize and incentivize repository engagement.

### Demographic and academic characteristics

Demographic factors such as gender, age, and academic qualifications also play a significant role in shaping deposit practices. Research suggests that women are more likely than men to deposit their work in both repositories and academic social networks ( Miguel et al., 2018). This trend may reflect broader patterns of engagement with collaborative and community-oriented tools. However, despite their higher participation rates, women deposit fewer documents on average than their male counterparts. This disparity could be linked to differences in research productivity, career stage, or access to resources ( Larivière et al., 2006).

Age and academic standing similarly influence deposit behaviours. Older researchers, as well as those with advanced degrees or senior positions, tend to deposit more documents on average ( Miguel et al., 2018). This trend may be explained by the cumulative effect of career length and experience, which likely provide these researchers with a greater volume of research outputs to share. Furthermore, individuals in higher academic ranks may have greater access to resources and a more nuanced understanding of the benefits of open access, driving their engagement with repositories and academic social networks ( Tmava & Ryza, 2023).

### Awareness and perception of repositories

The relationship between researchers’ understanding of open science principles and their sharing behaviours has emerged as a crucial area of investigation. Studies have demonstrated that comprehension of open access policies and copyright implications significantly influences repository usage patterns ( Tmava & Ryza, 2023). Research has shown that institutions investing in open science training and support services typically see higher rates of repository adoption and more sophisticated sharing strategies among their researchers ( Quinn, 2023). Moreover, studies have indicated that researchers with greater awareness of open science principles are more likely to engage in strategic version sharing and utilize multiple platforms effectively ( Tmava & Ryza, 2023).

Awareness and perception of repositories significantly impact researchers’ willingness to engage with these platforms. Many researchers demonstrate a preference for academic social networks like ResearchGate, often citing the advantages these platforms offer in terms of visibility, networking, and user-friendly interfaces. By contrast, institutional repositories are frequently underutilized due to a lack of awareness or understanding of their potential benefits ( Borrego, 2017). This lack of engagement can also be attributed to perceived complexity or limited training in the use of repository systems.

Researchers who are already predisposed to open access principles tend to adopt more proactive deposit behaviours, utilizing both institutional repositories and academic social networks. Their commitment to openness and broad dissemination of knowledge drives their engagement with multiple channels ( Eva & Wiebe, 2019). However, for researchers less familiar with open access or uncertain about its implications, the perceived advantages of academic social networks may overshadow the benefits of institutional repositories ( Borrego, 2017). These findings highlight the critical need for targeted awareness campaigns and training programs aimed at enhancing researchers’ understanding of repository systems and encouraging their adoption.

### Institutional context and policy impact

The influence of institutional frameworks on researcher behaviour has been extensively documented in the literature. Studies have demonstrated that well-designed institutional mandates, supported by robust infrastructure and clear guidelines, can significantly increase repository usage and compliance rates ( Quinn, 2023). However, research has also revealed considerable variation in policy effectiveness, with success often depending on factors such as enforcement mechanisms, support services, and institutional culture ( Bratt, 2023b, 2023a). Studies examining policy implementation have highlighted the importance of aligning institutional requirements with disciplinary practices and researcher workflows ( Kodua-Ntim, 2023).

The availability of infrastructure and resources is a pivotal factor influencing researchers’ ability to deposit their work. In resource-constrained environments, particularly in regions such as Tanzania, poor internet connectivity, low bandwidth, and unstable power supplies create significant barriers to the use of institutional repositories ( Mbughuni et al., 2022). These infrastructural limitations not only hinder access but also discourage researchers from engaging in repositories, as the process may be perceived as overly cumbersome or unreliable.

Conversely, institutions that invest in robust infrastructure and provide comprehensive support for data management and deposit routines, mainly through libraries’ actions, can significantly enhance researchers’ participation ( Bratt, 2023b; Ishak et al., 2019). Access to reliable internet, efficient repository systems, and adequate training opportunities empower researchers to deposit their work more effectively. Institutions that prioritize these aspects are likely to see higher engagement rates, particularly among early-career researchers or those who might otherwise face technological challenges ( Ishak et al., 2019).

### Implications

The variables influencing deposit practices in institutional repositories and academic social networks are multifaceted, encompassing disciplinary norms, institutional culture, demographic factors, and awareness of repository benefits; and there are still very unexplored, as there are few studies about it. The interplay between these potential factors underscores the complexity of fostering open access practices within the global research community. For example, researchers in resource-rich environments may exhibit high engagement with repositories due to robust infrastructural support, while those in resource-constrained settings face persistent challenges that limit their participation. Moreover, differences in disciplinary practices, academic standing, and demographic characteristics further shape the ways in which researchers interact with repositories and academic social networks.

Addressing these challenges requires a holistic approach that combines targeted interventions with broader structural changes. Efforts to improve infrastructure, particularly in under-resourced regions, are crucial for enabling equitable access to repository systems. At the same time, institutions must prioritize raising awareness about the benefits of repositories, dispelling misconceptions, and providing training to ensure that researchers are equipped to use these tools effectively. By addressing these factors, the scientific community can enhance deposit practices, promote open access, and ultimately contribute to a more inclusive and accessible research ecosystem.

## Methods

### Research design

This investigation employed a comprehensive quantitative research approach utilizing “Habits and perceptions regarding open science by researchers from Spanish Institutions” dataset ( Ollé-Castellà et al., 2023a), a cross-sectional survey methodology to examine deposit practices among researchers at Spanish institutions. The research design was constructed to capture a detailed snapshot of current practices and attitudes toward open science sharing across various academic contexts. Its approach allowed for the systematic collection of data regarding researchers’ behaviours, attitudes, and preferences while maintaining the ability to analyse relationships between multiple variables of interest.

The survey and subsequent dataset encompassed 666 responses from researchers affiliated with Spanish institutions, including universities and research centres, during a six-month data collection period from June 1, 2021, to December 1, 2021. The sampling strategy was designed to ensure representation across academic disciplines, career stages, and institutional types. The recruitment process utilized institutional communication channels, ensuring broad reach while maintaining institutional integrity ( Ollé-Castellà et al., 2023b). This approach facilitated access to a diverse population of researchers while working within established institutional frameworks.

The questionnaire of the “Dataset Habits and perceptions regarding open science by researchers from Spanish Institutions” was developed through a process of design and validation, with versions prepared in both Spanish and English to ensure maximum accessibility and precise data collection. The questionnaire architecture was structured to progress logically through several key domains of investigation. The demographic section gathered essential information about respondents’ characteristics, including age categorizations (21-35, 36-50, 51-65, over 65), gender, institutional affiliation, and academic discipline ( Ollé-Castellà et al., 2023a).

The instrument’s core sections addressed knowledge and practices related to open science, incorporating questions about repository usage patterns and platform preferences for different types of scholarly materials. The survey employed five-point Likert scales for measuring attitudes and frequencies. Questions about article deposit behaviours explored the types of versions shared, deposit frequency, and platform choices, while the dataset sharing section investigated repository usage patterns and sharing practices.

Data collection process was executed through electronic distribution strategy. Initial contact with potential participants occurred through institutional channels, with the distribution package including comprehensive study information, informed consent documentation, and secure access to the online survey platform ( Ollé-Castellà et al., 2023b).

### Analysis methods

The analytical strategy for this study was designed to systematically examine the complex relationships between researchers’ characteristics and their deposit behaviours across multiple dimensions. Our analysis framework incorporated several complementary statistical approaches to address the research objectives comprehensively.

Prior to analysis, data was screened for missing values, and response patterns were examined to ensure data quality. Variables were coded and transformed as necessary to meet the assumptions of the planned statistical tests, as it is shown below. All analyses were conducted using IBM SPSS Statistics version 27, after converting.csv original dataset file into an equivalent.sav file.

Then, examination of deposit practices followed a three-tiered analytical approach. First, we conducted a series of chi-square tests of independence to examine the relationships between categorical variables, particularly focusing on hints for associations between demographic characteristics and deposit behaviours to be further analysed. Second, for in depth comparing deposit rates across different categories in each factor, we employed independent samples Bonferroni t-tests.

For that, a custom table was created to show columns percentage and Bonferroni proportions comparison test significance. Variables in columns were: Open Science Awareness (transformed from P128 in original dataset as follows: 0 through 1 = 1 ‘No’ and 2 through 3 = 2 ‘Yes’, Gender (p134 in original dataset), Age group (P135 in original dataset), Discipline (P141 in original dataset) and Type of Institution (transformed from P136 trough P140 in original dataset as follows: if ((P136 = 1 OR P138 = 1) AND P137 = 0 AND P139 = 0) = 1 ‘Public’; if ((P137 = 1 OR P139 = 1) AND P136 = 0 AND P138 = 0) = 2 ‘Private’; if ((P136 = 1 AND P137 = 1) OR (P137 = 1 AND P138 = 1) OR (P138 = 1 AND P139 = 1)) = 3 ‘Mixed’; and if (P140 was not equal to 0) = 4 ‘Other’.

Variables in rows were: P2R ‘Number or Open Access articles published in 2019-2020’ (transformed from P2 as follows: (2 = 0 ‘No article deposit’) (1 or 3 or 4 = 1 ‘Article deposit done’) (5 = 9 ‘No paper in last 2 years’)), P3 ‘Deposit in repository, published version’, P4 ‘Do not deposit published articles’, P5 ‘Deposit in repository, accepted version’, P6 ‘Deposit in repository, pre-print version’, P7 ‘Deposit in academic networks, published version’, P8 ‘Deposit in academic networks, accepted version’, P9 ‘Deposit in academic networks, pre-print version’, P73R (transformed from P73 as follows: (0 or 6 = 0 ‘No data deposit’) (1 = 1 ‘Data deposit done’)), P74 ‘Deposit data in institutional repository’, P75 ‘Deposit data in funder’s repository’, P76 ‘Deposit data in thematic repository’, P77 ‘Deposit data in general repository’, P78 ‘Deposit data in research data repository’ and P79 ‘Deposit data in data journals’.

Third and last, to control confounding variables and to examine the interplay between multiple factors identified as significant and non-significant in bivariate analyses, we conducted multiple regression analysis. A crucial component of this regression model was the development of a composite article index and data deposit index. These indexes were constructed by aggregating responses from six questionnaire items related to article deposit practices, resulting in a score ranging from 0 to 6; and by aggregating responses from four questionnaire items related to data deposit practices, resulting in a score ranging from 0 to 4. The indexes were designed to capture the breadth and intensity of researchers’ deposit behaviours across different platforms and version types. The internal consistency of these indexes was assessed using Cronbach’s alpha to ensure reliability. Thus, regression models use article and data deposit (as binary and as metric variables) as dependent variables and incorporated all independent variables of interest transformed as it follows:

- Knowledge about open science (recoded from P128, that had four categories, into a binary variable: “no”, first two categories, and “yes”, two other categories).
- Gender (recoded from P134 into a binary variable: “male” or “female/other”).
- Discipline (as in original dataset, P141).
- Type of institution (from original variables P136 ‘Work in public university’, P137 ‘Work in private university’, P138 ‘Work in public research institution’ and P139 ‘Work in private research institution’ into a new binary variable: “public”, if only yes was marked in P136 and/or P138, or “other”, for the rest of options).

The regression models were assessed for standard assumptions including linearity, normality of residuals, homoscedasticity, and multicollinearity. The analysis protocol included specific procedures for handling missing data: Cases with missing values were excluded in analyses where complete data were not available for all variables of interest. This approach was chosen to maximize the use of available data while maintaining the integrity of the analysis.

This levelled and comprehensive analytical framework enabled us to examine the complex patterns of deposit behaviours while accounting for multiple influential factors and their interactions. The approach allowed for both a broad understanding of general patterns and detailed examination of specific relationships, providing a solid foundation for addressing study’s research objectives.

## Results

The analysis of deposit practices reveals interesting patterns in data sharing behaviours across different demographic and institutional variables. The results, presented in detail below, show the role of individual characteristics, institutional contexts, and disciplinary norms in shaping open science practices, and can be replicated using extended data files. These findings are organized into major sections examining article deposit practices and dataset sharing patterns. The main statistical results, regression models’ results, are shown in Table 1 and commented in the following sections, while results of chi square tests and Bonferroni t-tests of categories’ proportions are just specified in text, due to the huge size of those tables.

**Table 1.** Results for regression models.

Variable	Article deposit *dychotomous* (Exp(B))	Data deposit *dychotomous* (Exp(B))	Article deposit *index* (Beta)	Data deposit *index* (Beta)
Knows what Open Science is	1.883 ** (.005)	1.846 ** (.002)	0.142 ** (.001)	0.091
Gender = Male	1.629 * (.029)	1.952 ** (.001)	0.124 ** (.003)	-0.088
Gender = Female and others (reference)	#	#	#	#
Discipline = Arts and Humanities	3.990	1.349	0.161 *** (.000)	-0.05
Discipline = Life Sciences	2.775 * (.012)	3.156 *** (.000)	0.2 *** (.000)	0.181 * (.013)
Discipline = Experimental Sciences	3.028 * (.011)	1.590	0.15 ** (.001)	0.061
Discipline = Social Sciences	0.927	2.325 * (.013)	0.115 ** (.008)	0.091
Discipline = Engineering	0.841	1.658	0.062	-0.041
Discipline = Mathematics and Physical Sciences	1.074	2.129	0.102 * (.020)	0.349 *** (.000)
Discipline = Health Sciences (reference)	#	#	#	#
Type of center = Public	0.966	0.579 * (.043)		0.01
Type of center = Private or mixed (reference)	#	#	#	#

Notes: “#” indicates reference category. Significance levels:

* p < 0.05,

** p < 0.01,

*** p < 0.001.

### Article deposit practices

*Previous Knowledge and Awareness of Open Science*

Knowledge about open science emerged as a crucial factor in determining researchers’ deposit behaviours. Scholars who reported being familiar with open science principles demonstrated significantly higher rates of depositing scientific articles in open access repositories (Bonferroni p < .001) compared to those with limited knowledge. This pattern was particularly evident in the deposit of both final published versions and preprints manuscripts, with the latter showing a moderate but significant difference (Bonferroni p < .045).

The impact of open science awareness extended beyond mere repository usage, influencing the timing and versioning of deposits, with knowledgeable researchers showing a plurality of strategies in their deposit practices. Bonferroni tests show significant higher prevalence of sharing accepted articles in public repositories (p = .045) and in academic social networks (p = .031), and preprints (p < .001). In general, regression analysis revealed that researchers with prior knowledge of open science principles were significantly more likely to engage in depositing different versions of their manuscripts at various stages of the publication process.

*Demographic patterns*

The analysis of gender-based differences in the practices of depositing academic articles in open access repositories or academic social networks reveals statistically significant patterns in multiple areas. These differences highlight varying behaviours between male and female and other gender identities researchers when it comes to open access dissemination of their work.

Significant differences were observed among researchers in sharing accepted and peer-reviewed versions of articles in open access repositories (Bonferroni p = 0.006) and academic social networks (Bonferroni p = .032), where male researchers show a higher prevalence. There is stronger evidence in the deposition of pre-prints (manuscripts prior to peer review) in open access repositories (Bonferroni p = .001) as well. There is no evidence supporting differences in depositing the published version of articles in open access repositories and academic social networks, or preprints in academic social networks.

Noticing that the same items were significantly different according to both factors, awareness of Open Science and gender, regression models could have given us some clues about a possible interaction between these two variables. However, in its results, awareness of Open Science principles and gender still proves to be significant in general article sharing, in both of its dependent variables’ versions.

The analysis of age-related patterns revealed a less important dynamic in repository usage. Researchers in the 21-35 age group showed a distinctive preference for depositing accepted manuscripts in academic social networks (Bonferroni p < .028) compared to their immediate older colleagues (36-50 age group). The raw data revealed that researchers over 65 showed less engagement, mainly because of their reduced number - or even existence - of papers to publish, though some categories lacked sufficient data for statistical comparison. This variable was excluded from final regression models as it showed no statistical significance, revealing evidence for questioning its link with article deposit practices.

In conclusion, data reveals that gender plays a role in the patterns of depositing academic work in open access platforms and academic social networks, as Spanish male researchers have a higher rate in depositing articles than female and other identities ones, while there is few to no evidence of age group as a factor.

*Disciplinary and institutional variations*

Some disciplinary variations emerged, thus revealing distinct scholarly communication cultures in Spain. Health Sciences researchers showed significantly lower rates of depositing accepted manuscripts compared to researchers from multiple other disciplines, including Life Sciences (Bonferroni p < .002), Experimental Sciences (Bonferroni p < .000), Social Sciences (Bonferroni p < .000), Engineering (Bonferroni p < .008), and Mathematics and Physical Sciences (Bonferroni p < .004).

Within the realm of academic social networks, disciplinary differences were equally pronounced. Health Sciences research also demonstrated significantly lower engagement with these platforms compared to Arts and Humanities (Bonferroni p < .000) and Social Sciences (Bonferroni p < .005) and Mathematics and Physical Sciences (Bonferroni p < .000). The last finding in this comparison is that Mathematics and Physical Sciences researchers demonstrated notably higher preprint deposit (Bonferroni p < .000).

This evidence needs to be nuanced, since regression models suggest that some disciplines may not be linked to article deposit, as some of the previous links were not significant in their interaction with the other variables of interest. Only Life Sciences and Experimental Sciences remain showing significant coefficients in both models. Thus, there is strong evidence that Health Sciences researchers deposit their scientific articles way less frequently, especially when comparing to these two fields.

Lastly, type of institutions does not show any significant relationship with article deposits in either analysis, Bonferroni proportions comparison tests or regression models.

### Dataset sharing practices

*Previous knowledge and awareness of open science*

The relationship between researchers’ knowledge about open science and their data deposit behaviours was examined through both Bonferroni-adjusted comparisons of column proportions and regression models. The proportion comparison tests revealed that knowledge of open science was significantly associated with the choice of deposit platforms and versions. Researchers who reported knowing what open science entails were more likely to prefer specific deposit platforms. However, these differences were not consistent across all measured outcomes. For instance, while knowledge about open science seemed to relate to certain behaviours, it did not always result in a uniform preference for specific platforms or versions.

In the regression models, knowledge of open science emerged as a key factor in predicting data deposit. The logistic regression model, which explored the likelihood of engaging in data deposit, showed a significant positive association (β = 1.846, p = 0.002). This indicates that individuals who were aware of open science principles were almost twice as likely to deposit their data compared to those without such knowledge. However, this awareness did not significantly influence the intensity of data deposit practices, as captured in the linear regression model (p > 0.05). This suggests that while knowing about open science promotes the act of depositing data, it does not necessarily lead to depositing it in multiple platforms, but just one.

*Demographic patterns*

As in the article deposit section, the analysis also examined the influence of demographic variables such as gender and age group on data deposit practices and platform preferences. From the Bonferroni-adjusted proportion comparisons for specific data deposit platforms, gender differences were not observed.

In the regression models, gender was a significant predictor of the likelihood of data deposit. The logistic regression model revealed that male researchers were significantly more likely to deposit data than female researchers or those in other gender categories (β = 1.952, p = 0.001). However, gender did not significantly affect the intensity of data deposit practices in the linear regression model, indicating that once the decision to deposit is made, gender does not play a substantial role in determining multiple deposits.

Age patterns, while explored in the proportion comparison analysis, showed no significant associations with platform preferences or version types in either the regression models or the proportion tests. This suggests that while there may be anecdotal or minor generational differences in data deposit practices, they do not emerge as statistically significant factors when controlling for other variables such as discipline and knowledge of open science.

*Disciplinary and institutional variations*

Disciplinary and institutional contexts showed the most consistent and significant patterns in the data. The Bonferroni-adjusted proportion comparisons revealed that researchers in Life Sciences were significantly more likely to engage with certain deposit practices and platforms compared to the reference category, Health Sciences. For example, Life Sciences researchers demonstrated significant associations with platforms that prioritize data openness and accessibility, as reflected in the strong statistical results (e.g., Life Sciences and deposit intensity, Bonferroni p = 0.013). Similarly, researchers in Mathematics and Physical Sciences were found to have strong associations with higher-intensity deposit practices, as seen in their linear model significant association with data deposit index (p < 0.001).

In the regression models, discipline emerged as a highly significant predictor of both the likelihood and intensity of data deposit. In the logistic regression model, researchers in the Life Sciences (β = 3.156, p < 0.001) and Social Sciences (β = 2.325, p = 0.013) were significantly more likely to deposit data compared to the reference category of Health Sciences. In the linear regression model, the intensity of data deposit practices, measured by data deposit index, was significantly higher for researchers in Mathematics and Physical Sciences (β = 0.349, p < 0.001) and Life Sciences (β = 0.181, p = 0.013). This indicates that these disciplines not only have higher participation rates in data deposit but also engage in more detailed or extensive data-sharing practices.

Interestingly, some disciplines, such as Arts and Humanities, did not show significant associations with either the likelihood or intensity of data deposit. This may reflect differing norms or expectations around data sharing in these fields, which traditionally rely less on quantitative datasets and more on interpretive or textual analysis.

Institutional type also played an important role. Researchers from public institutions were significantly more likely to engage in data deposit activities compared to those from private or mixed institutions. This trend was observed in both the proportion comparison tests and the regression models. The logistic regression model revealed that being affiliated with a public institution decreased the likelihood of depositing data (β = 0.579, p = 0.043). This finding aligns with the proportion comparisons, which indicated that private or mixed institution researchers consistently showed stronger preferences for depositing data on platforms.

Finally, the role of institutional type was reaffirmed in the linear regression analysis. Public institutions were associated with slightly lower scores on the data deposit index measure, though this result was not statistically significant in the linear model (p > 0.05). This suggests that while public institutions are more likely to encourage data deposit overall, their impact on the depth or intensity of deposit practices may be more variable and influenced by other factors, such as internal policies, available resources, infrastructure or individual discipline.

In sum, knowledge about open science and discipline emerged as the most significant predictors, influencing both the likelihood and intensity of data deposit. Gender was significant in predicting the likelihood of data deposit but did not affect its intensity, while age showed no consistent effects. Finally, researchers from public institutions and disciplines like Life Sciences and Mathematics displayed the highest engagement in data-sharing practices, underscoring the importance of institutional and disciplinary contexts in promoting open science.

### Integration of article and data deposit findings

The findings reveal complementary and, at times, contrasting patterns in article deposit and research data deposit practices, reflecting the interplay between individual characteristics, disciplinary norms, and institutional contexts in shaping scholarly communication behaviours. Together, these insights paint a nuanced picture of how open science principles are practiced in the academic community.

*The role of knowledge in open science*

Knowledge about open science consistently emerged as a crucial driver for both article and data deposits. Researchers who demonstrated awareness of open science principles were significantly more likely to engage in depositing articles at various stages of publication (preprints, accepted manuscripts, and published versions) and to utilize multiple deposit platforms. Similarly, knowledge was a strong predictor of the likelihood of depositing research datasets, as revealed by the logistic regression models for both practices (β = 1.846 for dataset deposit; β = significant for article deposit in both preprint and accepted manuscript versions).

However, the influence of open science knowledge differed between the two contexts. In article deposits, awareness not only increased the likelihood of participation but also encouraged diversity in platform and version choices. In contrast, for research datasets, knowledge strongly predicted the likelihood of deposit but did not necessarily lead to higher-intensity practices (e.g., depositing on multiple platforms). This difference suggests that while open science principles may broadly encourage openness, the practicalities of sharing data, which often involve discipline-specific repositories or specialized requirements, may temper the breadth of application.

*Demographic patterns: gender and age*

Gender differences were apparent in both article and research data deposits, though their influence varied in scope and intensity. Male researchers were more likely to deposit both articles and datasets, with significant findings in logistic regression models for both practices (β = 1.952 for dataset deposits). In article deposits, this gender gap extended to specific deposit types, including preprints and accepted manuscripts. The lack of significant gender differences in depositing final published versions of articles suggests that gender-based disparities are more pronounced in earlier stages of the publication process, where researcher discretion and initiative play a larger role.

Interestingly, while gender affected the likelihood of depositing datasets, it did not influence the intensity of data-sharing practices. Once engaged in data sharing, both male and female researchers demonstrated similar levels of activity. This pattern aligns with the findings for article deposits, where gender did not influence the preference for depositing in academic social networks. These results point to an initial barrier for some demographics in engaging with open science but suggest equal levels of participation once these barriers are overcome.

Age, on the other hand, played a relatively minor role in both article and research data deposits. While younger researchers (21–35 years) showed some preference for depositing accepted manuscripts in academic social networks, this trend was not statistically significant in regression models. Similarly, no significant age-related differences were observed in dataset deposit practices. This finding suggests that age, as an isolated factor, may be less important in shaping open science behaviours, especially when compared to more prominent variables such as discipline, institutional context, and knowledge about open science.

*Disciplinary and institutional influences*

Disciplinary norms and institutional contexts emerged as the most consistent predictors of both article and research data deposit practices. Health Sciences researchers were consistently less likely to engage in article and dataset deposits compared to their counterparts in Life Sciences, Mathematics, Physical Sciences, and Social Sciences. The regression models confirmed these patterns, with disciplines such as Life Sciences and Experimental Sciences showing strong positive associations with both the likelihood and intensity of deposit practices. In research data deposit, Mathematics and Physical Sciences researchers demonstrated the highest intensity scores, reflecting their discipline’s long-standing emphasis on reproducibility and transparency.

These disciplinary patterns highlight distinct scholarly communication cultures. For instance, researchers in Life Sciences and Experimental Sciences were significantly more likely to deposit both articles and datasets, suggesting that open science practices are deeply embedded in these fields. By contrast, Medical Sciences or Arts and Humanities researchers demonstrated lower levels of dataset deposit, aligning with their reliance on interpretive or textual analysis, which may not generate conventional datasets.

Institutional type was another critical variable, particularly in research data deposit. Researchers from public institutions were less likely to engage in data-sharing practices, as evidenced by both proportion comparisons and logistic regression models (β = 0.579 for likelihood of dataset deposit). This trend likely reflects stronger institutional mandates and resources for open science in public institutions. Interestingly, institutional type did not significantly influence article deposit practices, suggesting that the broader cultural and policy environment around data sharing may differ from that of article dissemination.

*Key contrasts and intersections*

While similar factors—knowledge, discipline, and institutional type—shaped both article and dataset deposit behaviours, the mechanisms and intensity of these influences differed. Article deposits were more strongly linked to individual agency, as reflected in the nuanced preferences for specific deposit versions and platforms. Dataset sharing, on the other hand, was more influenced by institutional and disciplinary structures, where specialized repositories and field-specific norms played a larger role.

## Discussion

### Knowledge of open science as a key driver of engagement

Awareness of open science principles emerged as a consistent and significant determinant of deposit practices for both articles and research datasets. Researchers who demonstrated a clear understanding of open science were more likely to engage with repositories and academic social networks, confirming the importance of knowledge in fostering engagement ( Miguel et al., 2018; Tmava & Ryza, 2023). The impact of awareness was particularly pronounced in article deposit practices, where it not only increased participation but also diversified platform use and version-sharing behaviours.

Interestingly, the influence of open science knowledge on data deposits was less clear. While knowledge positively influenced the likelihood of engaging in data deposit, it did not significantly affect the intensity or variety of deposits (e.g., the number of platforms used). This asymmetry may reflect the specialized nature of data repositories, where technical, ethical, and disciplinary considerations create additional barriers, even for well-informed researchers ( Asadi et al., 2019). These findings emphasize the need for targeted institutional support to complement awareness campaigns, particularly by addressing the complexities associated with data sharing.

### Gender differences: divergence from established patterns

The study’s findings regarding gender reveal a divergence from existing literature, which has often suggested that female researchers are more inclined toward collaborative and community-oriented open science practices ( Larivière et al., 2006; Miguel et al., 2018). Contrary to this narrative, male researchers in this study were more likely to deposit both articles and datasets, particularly at earlier stages of the publication process, such as preprints and accepted manuscripts.

This discrepancy invites several hypotheses. One possibility is that structural and contextual factors, such as disparities in workload or resource allocation, may disproportionately disadvantage female researchers in Spain, limiting their engagement with non-mandatory deposit practices. Research has highlighted that women in academia often face heavier administrative and teaching loads, which could constrain the time available for proactive activities like depositing preprints ( Stark et al., 2020).

Another hypothesis centres on differences in perceived risks and benefits associated with open science practices. Women may exhibit greater caution in depositing early-stage research due to concerns over visibility, quality perception, data protection or potential misuse, particularly in competitive academic environments ( Haddow, 2008). Alternatively, cultural and disciplinary norms specific to the Spanish academic context might amplify traditional gender dynamics, influencing the observed behaviours. These hypotheses align with Borrego (2017), who argued that gender disparities in repository usage might reflect broader systemic inequalities rather than intrinsic differences in open science attitudes.

Further investigation is warranted to disentangle these factors. Qualitative studies exploring researchers’ motivations and constraints could provide valuable insights into the gendered dynamics of open science practices, offering a nuanced understanding that goes beyond aggregate trends.

### The limited influence of age

In contrast to gender, age had little impact on deposit behaviours, questioning prior assumptions that younger researchers are more inclined to adopt open access practices due to their digital fluency and exposure to evolving scholarly norms ( Eva & Wiebe, 2019). While younger researchers in this study showed a slight preference for depositing accepted manuscripts in academic social networks, this trend did not achieve statistical significance in general.

This finding may indicate a levelling of engagement across generations in the Spanish context, potentially driven by uniform institutional mandates or the normalization of repository usage; or may be caused of methodological limitations, as the used dataset asked for group age and not just age as a linear variable. Nevertheless, findings here suggest that, while age might play a role in initial adoption phases, sustained engagement with open science may depend more on contextual factors, such as institutional support and disciplinary norms, than on generational differences.

### Disciplinary and institutional contexts: structural determinants of practice

As expected, disciplinary differences played a prominent role in shaping deposit behaviours, reaffirming the influence of field-specific norms and practices ( Borrego, 2017; Stark et al., 2020). Researchers in Life Sciences and Experimental Sciences were significantly more engaged in both article and data deposits, reflecting these fields’ emphasis on rapid dissemination and collaborative research. Conversely, researchers in the Health Sciences exhibited lower engagement, likely due to concerns over data confidentiality and restrictive publisher policies, as noted in previous studies ( Haddow, 2008).

These patterns align with Rogers’ ( Rogers, 2003) diffusion of innovation theory, which suggests that compatibility with existing workflows is a critical factor in adoption. Disciplines with more established norms for data sharing and preprints, such as Life Sciences, exhibit higher compatibility with open science practices, whereas fields like Health Sciences may perceive such practices as less aligned with their traditional frameworks.

Institutional type further influenced engagement, particularly in data-sharing practices. Researchers affiliated with public institutions were less likely to engage in data deposits compared to those in private institutions, despite public institutions’ explicit mandates favouring open science. This paradox may reflect resource disparities, protocols or administrative inefficiencies, such as insufficient funding for repository infrastructure or limited training programs, as highlighted by Mbughuni et al. (2022). Additionally, Spanish private institutions may place greater emphasis on compliance, offering stronger incentives for researchers to engage with repositories ( Ishak et al., 2019).

### Divergent dynamics in article and data deposits

The contrasting dynamics between article and data deposit practices illuminate the complexity of open science implementation. Article deposits appeared to be more strongly influenced by individual agency, with researchers exercising discretion over platform selection and version-sharing strategies. In contrast, data sharing was shaped more by systemic factors, such as institutional policies, technical requirements, and disciplinary norms.

This distinction highlights the need for differentiated policy interventions. While initiatives targeting individual behaviours, such as awareness campaigns and training programs, may suffice for article deposits, data-sharing practices require structural reforms. These could include investments in repository infrastructure, the development of standardized data-sharing protocols, and the integration of data-sharing requirements into funding mandates.

### Theoretical implications and policy recommendations

These findings extend theoretical discussions on the adoption of open science practices by emphasizing the interplay between individual and systemic factors. The differential impact of knowledge on article versus data deposits underscores the importance of tailoring open science initiatives to the unique affordances and constraints of each practice. Furthermore, the observed gender disparities challenge simplistic narratives about open science engagement, inviting a more nuanced exploration of how systemic inequalities shape scholarly communication behaviours.

From a policy perspective, the results point to several actionable strategies:

- Addressing Gender Disparities: Institutions should foster inclusive environments that reduce barriers for female researchers, such as providing administrative support and promoting equitable resource allocation.
- Enhancing Institutional Support: Investments in repository infrastructure, particularly in public institutions, could mitigate the resource-related barriers identified in this study.
- Standardizing Data-Sharing Protocols: Developing user-friendly, discipline-agnostic frameworks for data sharing could address the complexities that currently deter engagement.
- Disciplinary Tailoring: Policies must account for disciplinary differences, promoting flexible approaches that align with the unique norms and practices of each field.

### Strengths and limitations

This study’s methodological design and sample of Spanish researchers provide a solid foundation for this analysis for understanding open science practices. However, the reliance on non-representative and self-reported data introduces potential biases, such as overreporting of desirable behaviours or underreporting of constraints. Additionally, the cross-sectional design limits causal inferences, necessitating longitudinal research to track the evolution of behaviours over time. Finally, the focus on Spain, while offering valuable context-specific insights, calls for comparative studies to explore how these findings generalize to other cultural and policy contexts.

#### Ethical considerations

No human or committee consent was required as data comes from a third party. Original data has CC BY license. Original data was not suitable for ethical approval committee decision as no identity or personal data was asked or collected. Repository ensures, to the extent possible, that data are created, curated, accessed, and used in compliance with disciplinary and ethical norms. Contact was made by email, after scrapping WoS database in search for Spanish authors. Survey was anonymous and consent was given on the platform before participating ( Ollé-Castellá et al., 2023b). Alterations related in Methods section have not distorted scientific meaning.

## Data Availability

CORA: Dataset sobre hábitos y percepciones sobre la ciencia abierta de investigadores de instituciones españolas. https://doi.org/10.34810/DATA690 ( Ollé-Castellà et al., 2023a). The project contains the following underlying data:
• File name: respostes_personal_investigador_2023_04_11.csv. Description: Responses to a survey among Spanish researchers. (Anonymised answers to questionnaire, 666 rows, 142 columns). • File name: Cuestionario_personal_investigador_ciencia_abierta.odt. Description: Survey instrument (questionnaire) among Spanish researchers in Spanish. • File name: Survey_researchers_open_science_english_version.odt. Description: Survey instrument (questionnaire) among Spanish researchers in English. Data are available under the terms of the Creative Commons Attribution 4.0 International license (CC-BY 4.0). Analysis code available from: https://github.com/Chaniwaldo/Analysis-code-OS-deposit-in-Spain Archived analysis code at time of publication: https://doi.org/10.5281/ZENODO.14615980 ( de Gracia, 2025). License: Apache 2.0 *STROBE guidelines* *checked.*
