# Supplementary material for:  Open Science in Spain: Influence of Personal and Contextual Factors on Deposit Patterns
Source: F1000Res. 2026 Sep 30;14:212. Originally published 2025 Feb 14. [Version 2] doi: 10.12688/f1000research.160207.2 (PMC13662382; doi:10.12688/f1000research.160207.2)
Supplement: Peer review history

# Reviewer report: Reviewer response for version 2

**DOI:** 10.5256/f1000research.211468.r526091

**Published:** 2026-10-13

**Authors:** Roxana Cerda-Cosme

**Affiliations:** 1 Universidad Carlos III de Madrid, Madrid, Spain

The article examines associations between personal and contextual factors and self-reported article and data deposit practices among researchers affiliated with Spanish institutions. It reuses a survey of 666 participants conducted between June and December 2021, whose results were published by Ollé and colleagues in 2023. The factors examined include gender, age, knowledge of open science, discipline, and institution type.

 The topic is relevant, but the manuscript needs to explain its contribution more clearly, update the policy context, and keep its interpretations within the available evidence. This review does not include a specialist assessment of the statistical procedures.

 1. Contribution and time scope

 It is unclear what the manuscript adds to the study by Ollé and colleagues. Several conclusions and recommendations, particularly those concerning training and institutional support, overlap with the original publication. The authors should identify which additional questions they answer, and which findings extend, qualify, or differ from the earlier results.

 The findings describe practices reported in 2021, rather than the current situation of open science in Spain. The authors should make this time scope clear and acknowledge later changes. Reusing the same dataset does not provide independent confirmation or allow changes in practices over time to be assessed.

 Repeating the survey under comparable conditions would be a useful direction for future research.

 2. Policy framework

 The introduction presents Horizon 2020 requirements as part of the current European framework. It should distinguish these from Horizon Europe (2021–2027) and update the information on open access, deposit, licenses, trusted repositories, and FAIR data management.

 Horizon 2020 can remain as background and as the framework for projects funded under that programme. The authors should distinguish policies relevant to the study period from later developments, deposit deadlines from access embargoes, and programme-specific obligations from requirements applying to researchers in Spain more generally.

 The names of the Spanish laws should also be corrected, and the required manuscript versions and deposit deadlines should be clearly stated for each framework.

 3. Spanish context

 The literature review needs a summary of Spanish studies related to the factors examined. This would help identify existing knowledge, the gap addressed by the article, and the studies against which its findings can be compared.

 International studies can provide context, but their relevance should be explained. For example, connectivity and electricity supply problems reported in Tanzania should not be applied to Spain without supporting evidence. Where Spanish studies on a particular factor are lacking, this should be stated.

 4. Objectives

 The authors should distinguish descriptive objectives from those examining associations, using specific objectives or research questions.

 They should also clarify which aspects concern articles and which concern data, since manuscript versions apply to articles. The term “preferences” should be used for preferences measured directly, rather than inferred from reported practices.

 5. Repositories and academic social networks

 The manuscript should define institutional and subject repositories and academic social networks, explaining their functions, benefits, and differences in preservation, metadata, interoperability, persistent identifiers, and access and reuse conditions.

 Sharing a publication on ResearchGate does not automatically mean depositing it in a repository, meeting a mandate, or ensuring open access. Using several channels also does not, by itself, demonstrate a stronger commitment to open access.

 These distinctions should be maintained when interpreting the results. Publication, sharing, and deposit should not be used as interchangeable terms.

 6. Statements about gender

 The hypothesis that women are more cautious about sharing research at an early stage needs specific supporting evidence. Concerns about visibility, quality, data protection, or misuse can affect researchers of different genders. Reported deposit practices do not establish these motivations.

 The reference to Haddow (2008) should be checked. Its published description concerns disciplinary differences, assisted deposit, and mandates, without providing specific evidence of greater caution among women.

 The authors should also distinguish research output, participation in deposit, and the range of sharing practices. Conclusions should match the aspect measured and remain limited to the study sample.

 Career stage, leadership, and decision-making roles within teams may be proposed as alternative explanations but should not be presented as mechanisms demonstrated by this study.

 7. Conclusions and recommendations

 The manuscript presents some explanations as hypotheses, which is appropriate. This caution should be maintained throughout the discussion and recommendations, and the hypotheses should be supported by relevant references.

 The distinction between individual autonomy in article deposit and systemic factors in data sharing needs stronger justification. The study also does not demonstrate that awareness campaigns and training are sufficient to improve article deposit.

 The recommendations should acknowledge that some data management and open-access requirements already exist in funding mandates. The authors should distinguish the existence of these requirements from their implementation and focus their proposals on support, resources, and monitoring, without presenting these needs as directly established findings.

Is the work clearly and accurately presented and does it cite the current literature?

Yes

If applicable, is the statistical analysis and its interpretation appropriate?

I cannot comment. A qualified statistician is required.

Are all the source data underlying the results available to ensure full reproducibility?

Yes

Is the study design appropriate and is the work technically sound?

Yes

Are the conclusions drawn adequately supported by the results?

Partly

Are sufficient details of methods and analysis provided to allow replication by others?

Yes

Reviewer Expertise:

Open science, research data management, open access, research data repositories, FAIR principles, scholarly communication.

I confirm that I have read this submission and believe that I have an appropriate level of expertise to confirm that it is of an acceptable scientific standard, however I have significant reservations, as outlined above.

# Reviewer report: Reviewer response for version 1

**DOI:** 10.5256/f1000research.176060.r368172

**Published:** 2025-03-18

**Authors:** Blanca Rodriguez-Bravo

**Affiliations:** 1 Department of Library and Information Science, University of León, León, Castile and León, Spain

The paper gives information about the factors that influence open science practices and takes into account the different variables that can be studied. The results are of interest.

 The methodology seems correct although I have limitations to judge the statistical methods used.

 I only miss a more comprehensive analysis of the literature that may have enriched the literature review and the discussion.

Is the work clearly and accurately presented and does it cite the current literature?

Partly

If applicable, is the statistical analysis and its interpretation appropriate?

I cannot comment. A qualified statistician is required.

Are all the source data underlying the results available to ensure full reproducibility?

Yes

Is the study design appropriate and is the work technically sound?

Yes

Are the conclusions drawn adequately supported by the results?

Yes

Are sufficient details of methods and analysis provided to allow replication by others?

Yes

Reviewer Expertise:

Scholarly communication; Library and Information Science

I confirm that I have read this submission and believe that I have an appropriate level of expertise to confirm that it is of an acceptable scientific standard.

# Reviewer report: Reviewer response for version 1

**DOI:** 10.5256/f1000research.176060.r367060

**Published:** 2025-03-06

**Authors:** Juan-José Boté-Vericad

**Affiliations:** 1 Universitat de Barcelona, Barcelona, Catalonia, Spain

**Summary**

 This study explores the factors influencing the deposit of academic publications and research data in open-access repositories by Spanish researchers. Using survey data from Spanish academics, it examines personal attributes (gender, age, knowledge of open science) and contextual variables (academic discipline, institutional type) that affect deposit behaviors. The study employs statistical analyses, including chi-square tests, Bonferroni-adjusted comparisons, and regression models, to identify patterns and relationships. The findings highlight significant disciplinary and institutional differences, as well as gender disparities in deposit practices. The study contributes to open science discourse by offering insights into policy implications and practical recommendations for enhancing repository use in Spain.

 1. Is the work clearly and accurately...?

 The manuscript is well-structured and provides a clear presentation of research objectives, methods, results, and implications. The literature review is comprehensive and cites relevant studies on repository use, open science adoption, and institutional policies.

 2. Is the study design appropriate...?

 The study employs a robust quantitative design, drawing on a well-structured dataset. The use of chi-square tests, Bonferroni comparisons, and multiple regression analyses ensures a rigorous approach to examining deposit patterns.

 3. Are sufficient details of methods and... ?

 The methods section is highly detailed, with thorough documentation of dataset transformations, statistical tests, and analytical procedures. However, some descriptions are overly complex, making it difficult for non-specialists to follow. The transformations of dataset variables should be presented in a more structured format (e.g., using a table or appendix) to improve clarity. Additionally, while the study mentions checking for regression assumptions (e.g., normality, homoscedasticity), a brief summary of diagnostic results should be included to confirm statistical validity.

 4. 4. If applicable, is the statistical analysis... ?

 The statistical methods are appropriate for the research questions and data type.

 5. Are all the source data underlying... ?

 The study provides access to the dataset (CORA repository) and analysis code (GitHub and Zenodo). However, clearer guidance on how to use the dataset for replication would be beneficial. A brief explanation of how to access and interpret the raw data should be included in the *Data Availability* section. Additionally, while the analysis code is available, it would be useful to specify whether it includes all necessary steps to replicate the study’s findings.

 6. Are the conclusions drawn... ?

 The conclusions align with the study’s findings, particularly regarding the role of open science knowledge, disciplinary differences, and gender disparities. However, the discussion could further elaborate on:

- Why public institutions exhibit lower data deposit rates despite open-access mandates.
- Potential confounding factors influencing gender differences in deposit practices.
- The impact of methodological choices (e.g., age grouped as a categorical variable) on findings.

Additionally, the limitations section should explicitly acknowledge potential response biases and the cross-sectional nature of the data.

Is the work clearly and accurately presented and does it cite the current literature?

Yes

If applicable, is the statistical analysis and its interpretation appropriate?

Yes

Are all the source data underlying the results available to ensure full reproducibility?

Partly

Is the study design appropriate and is the work technically sound?

Yes

Are the conclusions drawn adequately supported by the results?

Partly

Are sufficient details of methods and analysis provided to allow replication by others?

Partly

Reviewer Expertise:

My area of expertise in Open Science and Research Data Management.

I confirm that I have read this submission and believe that I have an appropriate level of expertise to confirm that it is of an acceptable scientific standard, however I have significant reservations, as outlined above.

# Response

**Published:** 2026-09-25

**Authors:** Daniel de Gracia Palomera

**Affiliations:** QUIT-IET. Dpt of Sociology, Universitat Autonoma de Barcelona Departament de Sociologia, Bellaterra, Catalonia, Spain

We thank the reviewer for their careful reading of the manuscript and for their constructive comments, which we address point by point below. We would also like to apologise for the delay in answering to this review, which was due to personal circumstances on the part of this corresponding author.

 All changes made to the manuscript are to be included in version 2. We believe these revisions have improved the clarity, reproducibility, and analytical transparency of the paper. They are made in these sections:

**3. Are sufficient details of methods and analysis provided to allow replication by others?**

 We agree that the description of dataset transformations, presented previously only as continuous prose, was difficult to follow. We have therefore added a new Table 1, “Summary of variable transformations used in the analyses”, in the Methods section (Analysis methods), which lists, for every recoded variable used in the bivariate and regression analyses, the source variable(s), the recoding rule applied, and the resulting categories. A short sentence now directs the reader to this table at the point where the transformations are first described.

 Regarding regression diagnostics, we have re-run the assumption checks for both linear regression models (article deposit index and data deposit index) and added a concise summary of the results to the Methods section. Variance inflation factors were low for all predictors in both models (all VIF < 1.3), indicating no relevant multicollinearity. Residuals in both models showed a positive skew and departed from strict normality (Shapiro-Wilk p < .001) and homoscedasticity (Breusch-Pagan p < .001), which is consistent with the discrete, bounded nature of the two composite indices (ranges 0–6). We now state this explicitly and note that, because the complementary logistic regression models (which do not depend on these assumptions) point in the same direction as the linear models, we consider the substantive conclusions robust, while acknowledging that the linear-model coefficients should be interpreted with this caveat in mind.

**5. Are all the source data underlying the results available to ensure full reproducibility?**

 We have expanded the Data availability section to provide clearer replication guidance. We now explicitly state that users should download the raw survey export (respostes_personal_investigador_2023_04_11.csv) from the CORA repository and apply the archived analysis code, that includes a self-contained script that performs this conversion automatically from the raw file (and all the rest of the steps that allow results reproducibility), so that no manual recoding step or proprietary software is required.

 To further clarify that, we have also added a sentence to the Extended data paragraph clarifying that the archived Python code (GitHub and Zenodo, DOI 10.5281/ZENODO.14615980) covers the full analytical pipeline end-to-end — from the raw survey file to the final regression tables reported in Table 2 — and does not require SPSS or any other proprietary software to run as they are written in Python, addressing the reviewer’s request to specify whether the code includes all steps needed to replicate the study’s findings.

**6. Are the conclusions drawn adequately supported by the results?**

 We have expanded the Discussion in three places, and the Limitations section in one place, to address the specific issues raised by the reviewer:

 (a) Public institutions and data deposit: we have added a sentence to the “Disciplinary and institutional contexts” subsection noting that formal data-deposit mandates in Spanish public institutions are seldom accompanied by enforcement or monitoring mechanisms, so that compliance depends largely on individual researcher initiative rather than institutional oversight; this may weaken their practical effect relative to some private institutions, where repository use is more directly tied to internal evaluation, accreditation, or funding-renewal procedures.

 (b) Confounding factors in gender differences: we have added a sentence to the “Gender differences” subsection acknowledging that part of the observed gender gap may be confounded by unmeasured factors not captured by the survey instrument, such as seniority, contract type (temporary versus permanent), or caregiving responsibilities, which are known to be unevenly distributed by gender within the Spanish academic system, and we call for future studies to collect this information.

 (c) Age as a categorical variable: we have added a sentence to the “The limited influence of age” subsection explicitly discussing how treating age as a four-category ordinal variable, rather than as a continuous measure, reduces statistical power to detect smaller, gradual effects across the life course, and may partly explain the null findings reported for this variable.

 (d) Limitations: we have expanded the “Strengths and limitations” section to explicitly acknowledge that, because participation was voluntary and all measures were self-reported at a single point in time, the results may be affected by non-response bias and social-desirability bias, and that — combined with the cross-sectional design — the reported associations should be interpreted as descriptive of current practice rather than as evidence of causal or temporally ordered relationships.

 We hope these revisions, shown in V2 of the article, satisfactorily address the reviewer’s comments, and we thank again for the valuable feedback, which has strengthened the manuscript.
